# Risk Stratification for Patients with Chest Pain Discharged Home from the Emergency Department

**DOI:** 10.3390/jcm9092948

**Published:** 2020-09-12

**Authors:** Peter A. Kavsak, Joshua O. Cerasuolo, Shawn E. Mondoux, Jonathan Sherbino, Jinhui Ma, Brock K. Hoard, Richard Perez, Hsien Seow, Dennis T. Ko, Andrew Worster

**Affiliations:** 1 Department of Pathology and Molecular Medicine, McMaster University and Juravinski Hospital and Cancer Centre, 711 Concession Street Hamilton, Hamilton, ON L8V 1C3, Canada; 2 ICES McMaster, Faculty of Health Sciences, McMaster University, Hamilton, ON L8S 4K1, Canada; Joshua.Cerasuolo@ices.on.ca (J.O.C.); richard.perez@ices.on.ca (R.P.); seowh@mcmaster.ca (H.S.); 3 Division of Emergency Medicine, McMaster University, Hamilton, ON L8N 3Z5, Canada; shawn.e.mondoux@gmail.com (S.E.M.); sherbino@gmail.com (J.S.); worstea@mcmaster.ca (A.W.); 4 Department of Health Research Methods, Evidence and Impact, McMaster University, Hamilton, ON L8N 3Z5, Canada; maj26@mcmaster.ca; 5 McMaster University, Hamilton, ON L8N 3Z5, Canada; hoardb@mcmaster.ca; 6 ICES Central, Toronto, ON M4N 3M5, Canada; dennis.ko@ices.on.ca

**Keywords:** clinical chemistry score, high-sensitivity cardiac troponin, chest pain, emergency department, discharged, risk stratification

## Abstract

For patients with chest pain who are deemed clinically to be low risk and discharged home from the emergency department (ED), it is unclear whether further laboratory tests can improve risk stratification. Here, we investigated the utility of a clinical chemistry score (CCS), which comprises plasma glucose, the estimated glomerular filtration rate, and high-sensitivity cardiac troponin (I or T) to generate a common score for risk stratification. In a cohort of 14,676 chest pain patients in the province of Ontario, Canada and who were discharged home from the ED (November 2012–February 2013 and April 2013–September 2015) we evaluated the CCS as a risk stratification tool for all-cause mortality, plus hospitalization for myocardial infarction or unstable angina (primary outcome) at 30, 90, and 365 days post-discharge using Cox proportional hazard models. At 30 days the primary outcome occurred in 0.3% of patients with a CCS < 2 (*n* = 6404), 0.9% of patients with a CCS = 2 (*n* = 4336), and 2.3% of patients with a CCS > 2 (*n* = 3936) (*p* < 0.001). At 90 days, patients with CCS < 2 (median age = 52y (IQR = 46–60), 59.4% female) had an adjusted HR = 0.51 (95% confidence interval (CI) = 0.32–0.82) for the composite outcome and patients with a CCS > 2 (median age = 74y (IQR = 64–82), 48.0% female) had an adjusted HR = 2.80 (95%CI = 1.98–3.97). At 365 days, 1.3%, 3.4%, and 11.1% of patients with a CCS < 2, 2, or >2 respectively, had the composite outcome (*p* < 0.001). In conclusion, the CCS can risk stratify chest pain patients discharged home from the ED and identifies both low- and high-risk patients who may warrant different medical care.

## 1. Introduction

Now that high-sensitivity cardiac troponin (hs-cTn) assays are globally available and are considered the gold standard test for detecting myocardial injury, the utility of other cardiac biomarkers for investigating patients with chest pain in the emergency department (ED) has diminished [1,2,3,4,5,6]. In this regard, there has been much interest in applying different high-sensitivity cardiac troponin I (hs-cTnI) or high-sensitivity cardiac troponin T (hs-cTnT) concentration cutoffs for risk stratification [6,7,8]. However, a limitation of this approach, is that it relies solely on the measurement of a single analyte (hs-cTnI or hs-cTnT), and as such is subject to the well-documented analytical issues of these assays affecting the accuracy and reproducibility of the results [2,9,10,11]. Moreover, there are other common biomarkers (e.g., glucose and creatinine), besides cTn, that have important pathophysiological roles in acute coronary syndrome (ACS) and might influence risk stratification [12,13,14].

We have previously derived and validated a simple clinical chemistry score (CCS) at ED presentation that has superior diagnostic performance than either hs-cTnI or hs-cTnT alone for myocardial infarction (MI) [15]. Briefly, the CCS is a six-category score (0 to 5) with points derived from different levels of glucose, the estimated glomerular filtration rate (eGFR), and hs-cTnI or hs-cTnT using established cutoffs [15]. The diagnostic performance of the CCS has been validated in different settings and using different manufacturers’ assays [16,17,18]. The objective for the present study was to assess whether the CCS would provide additional risk stratification information for chest pain patients discharged home from the ED, which are overall a very low risk group of individuals with a reported composite outcome of all-cause death, MI or unstable angina (UA) at 30 days being ≤ 1.0% in the province of Ontario, Canada [19].

## 2. Methods

### 2.1. Study Design and Population

**Figure 1:**
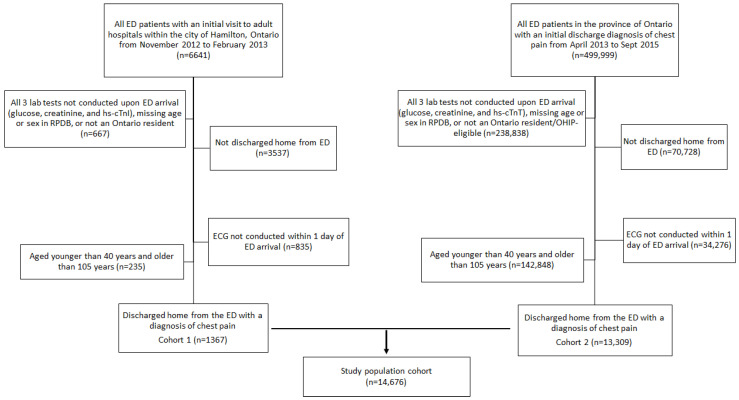
Flow diagram of study population cohort. ED: emergency department; hs-cTn: high-sensitivity cardiac troponin; RPDB: registered persons database; OHIP: Ontario health insurance plan; ECG: electrocardiogram.

The study population of 14,676 ED patients with chest pain was comprised of two different cohorts: cohort 1 included 1367 patients who underwent hs-cTnI testing (Abbott Diagnostics) and cohort 2 included 13,309 patients who underwent hs-cTnT testing (Roche Diagnostics) (see Figure 1 for flow diagram). For cohort 1, consecutive ED patients (first presentation from 28 November 2012 to 28 February 2013) who were investigated for possible ACS at three hospitals (teaching hospitals) in the city of Hamilton, Ontario, Canada and had cardiac troponin results were selected (*n* = 6641) [20]. Patients were excluded if they did not have glucose results, creatinine results (for eGFR calculation via CKD-EPI equation), Abbott hs-cTnI results, or missing information which prevented linkages to databases (*n* = 667), were not discharged home (*n* = 3537), did not have an electrocardiogram (ECG) conducted (*n* = 835), or were younger than 40 years or older than 105 years (*n* = 235), leaving 1367 patients discharged home from the ED with a diagnosis of chest pain [19,20,21].

For cohort 2, all ED patients (first presentation between April 2013 to September 2015) in the province of Ontario, Canada discharged from the ED with a diagnosis of chest pain (*n* = 499,999) were selected [21]. Patients were excluded if they did not have glucose and creatinine results (for eGFR calculation via CKD-EPI equation [22]), Roche hs-cTnT results (laboratory data obtained from the Ontario Laboratory Information System) or had missing information which prevented linkages to databases (*n* = 238,838), were not discharged home (*n* = 70,728), did not have an ECG conducted (*n* = 34,276), or were younger than 40 years or older than 105 years (*n* = 142,848), leaving 13,309 patients discharged home from the ED (3 community hospitals, 1 small hospital and 5 teaching hospitals) with a diagnosis of chest pain [19,21]. The final study population consisted of patients from both cohorts of whom had been discharged home, had all necessary information completed, and fell within the correct age range, leaving 14,676 suitable patients. Of note, the age range of 40 to 105 years as well as the requirement for an ECG was to identify a population more likely to have symptoms/pain related to cardiac origin (see Supplement for description of cohort 1 and cohort 2).

### 2.2. Clinical Chemistry Score (CCS)

The CCS consists of the sum of points obtained when using an algorithm that requires the earliest glucose level, eGFR level, and hs-cTnI or hs-cTnT levels for the derivation of the scores [15]. Briefly, the scores are generated for each patient as follows: if glucose level ≥ 5.6 mmol/L, then assign 1 point (if below then no points); if eGFR < 90 mL/min/1.73 m^2^, then assign 1 point (if above then no points); if hs-cTnI level < 4 ng/L = 0 points or 4–14 ng/L = 1 point or 15–30 ng/L = 2 points or >30 ng/L = 3 points; or if hs-cTnT level < 8 ng/L = 0 points or 8–18 ng/L = 1 point or 19–30 ng/L = 2 points or >30 ng/L = 3 points [15]. The final CCS was the sum of the points from glucose, eGFR and hs-cTnI or hs-cTnT derived from patients from both cohorts.

### 2.3. Outcomes and Statistical Analysis

The primary outcome was the composite of all-cause death, MI or unstable angina (UA) as previously described [19]. Secondary outcomes included death/MI alone and major adverse cardiac events (MACE; defined as the composite of death/MI/UA/percutaneous coronary intervention [PCI] or coronary artery bypass grafting [CABG]) [15,21]. Administrative and clinical databases housed at the Institute for Clinical Evaluative Sciences (ICES, Toronto, Ontario, Canada) via unique encrypted patient identifiers were used to obtain past medical history and outcomes. The Ontario Registered Persons Database (RPDB) contained all information on patient demographics and death date. All inpatient hospital discharges were captured in the Canadian Institute of Health Information (CIHI) Discharge Abstract Database (e.g., MI from diagnostic codes in the hospitalization database which is available for all patients in Ontario). We used the Ontario Health Insurance Plan (OHIP) database to capture all physician billings and outpatient visits. These datasets were linked using unique encoded identifiers and analyzed at ICES. Outcomes were evaluated at 30, 90, and 365 days [19,20,21]. The analyses were from the patients’ earliest record with all subsequent ED visits excluded (i.e., unique patient only represented once in the analysis).

A miss event rate of 1% for an acute ischemic event/death for discharged ED patients at 30 days is considered by several emergency medicine groups to be acceptable [23,24]. Accordingly, we set the score from the CCS that achieved close to 1% event rate at 30 days as the referent group. Scores below and above were grouped together to yield three different groups: low CCS, referent, and high CCS. Among these three groups we compared baseline characteristics (demographic and clinical) with a five-year look-back across these categories. Kaplan–Meier survival curves were constructed for the three CCS categories and log-rank test was used to compare the survival difference between these categories for the primary and secondary outcomes. We also performed Cox proportional hazard modeling for the primary outcome (model 1 unadjusted, model 2 adjusted for age and sex and model 3 adjusted for age, sex, past medical history of arrhythmia, heart failure, diabetes, hypertension, MI, peripheral vascular disease, renal disease, stroke and UA) to assess the hazard ratio (HR) of CCS categories with and without adjustment for confounders. For the secondary outcomes model 3 was further adjusted for history of PCI and CABG. The negative predictive values (NPVs) were determined for 30 day MACE for CCS < 2 and hs-cTn < 5 ng/L, as a common cutoff level of 5 ng/L for both the Roche and Abbott hs-cTn assays has been proposed [25]. NPV estimates ≥ 99.5% were deemed acceptable [26]. Statistical analyses (e.g., means compared using ANOVA, medians using Kruskal–Wallis test, categorical variables using chi-squared test, Cox modelling, c-statistic, Kaplan–Meier and log-rank analyses) were performed using SAS 9.1.3 software (SAS Institute Inc, Cary, NC, USA) while NPVs were calculated using MedCalc Statistical Software version 19.4.0 (MedCalc Software Ltd., Ostend, Belgium). The Hamilton Integrated Research Ethics Board approved this study (project number: 4717-D, date: March 12, 2018).

## 3. Results

The overall study population cohort (*n* = 14,676) median age (interquartile range; IQR) was 59 years (50 to 71) with 55.1% of the patients being female. Only a third (33%) of the study population had a follow-up with a cardiologist within 30 days following the ED discharge (Table 1). At 30 days following ED discharge, there were 147 unique patients that had the primary outcome (1.0% of total), with patients with a CCS = 2 (*n* = 4336, median age [IQR] = 62 years [52 to 71], 55.3% females) having 37 instances of the composite outcome (0.9%) (set as the referent group). Patients with a CCS < 2 (*n* = 6404, median age [IQR] = 52 years [46 to 60], 59.4% female) had fewer composite outcomes (*n* = 18; 0.3%) while patients with a CCS > 2 (*n* = 3936, median age [IQR] = 74 years [64 to 82], 48% females) had more outcomes (*n* = 92, 2.3%) as compared to the CCS = 2 reference group (*p* < 0.001) (Table 1). The median (IQR) hs-cTnI and hs-cTnT concentrations for the CCS < 2, 2, > 2 groups were 2 ng/L (1–3) and 5 ng/L (3–6), 3 ng/L (2–5) and 7 ng/L (5–13), 9 ng/L (6–16) and 14 ng/L (12–24), respectively (see Supplemental Tables S1 and S2).

At 30 days there were 42 deaths (0.3%), 58 MIs (0.4%), 97 death/MIs (0.7%) and 216 patients that experienced MACE (1.5%). The NPVs for 30-day MACE was 99.6% (95% CI: 99.4 to 99.7) for CCS < 2 and 99.7% (95% CI: 99.5 to 99.8) for hs-cTn < 5 ng/L. The proportion of patients classified as low risk was higher for the CCS < 2 (43.6%) as compared to hs-cTn < 5 ng/L (28.1%) (difference = 15.5%; 95%CI: 14.4 to 16.6) (*p* < 0.001). Of the 6404 patients with a CCS < 2, only 0.4% had a hs-cTn level > 14 ng/L, while for the 3936 patients with a CCS > 2, only 1.7% had a hs-cTn level < 5 ng/L.

**Figure 2:**
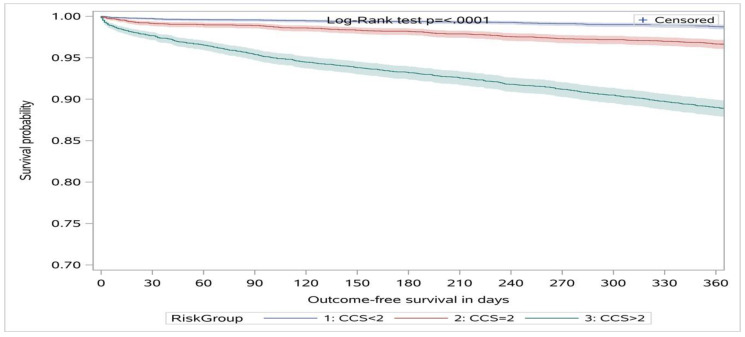
Kaplan–Meier survival curves for the primary outcome (i.e., all-cause death, MI or UA) over 365 days stratified by CCS < 2, CCS = 2, and CCS > 2. CCS: clinical chemistry score.

The unadjusted and adjusted HRs for the CCS < 2 were significantly lower for the primary outcome as compared to the CCS = 2 group (i.e., CCS < 2 was associated with a 60% decreased risk of having the composite outcome compared to CCS = 2 over 30 days) while the CCS > 2 had significantly higher HRs for the primary outcome (i.e., CCS > 2 was associated with a 2-fold increased risk of having the composite outcome compared to CCS = 2 over 30 days) (*p* < 0.01) (Table 2, see Supplement for HRs in Cohort 1 (Table S3) and Cohort 2 (Table S4) for the primary outcome). For the secondary outcomes at 30 days, the adjusted HRs were 0.39 (95%CI: 0.18 to 0.84) for CCS < 2 and 2.88 (95%CI: 1.67 to 4.97) for CCS > 2 for death/MI (c-statistic = 0.80) and 0.34 (95%CI: 0.22 to 0.56) for CCS < 2 and 1.74 (95%CI: 1.24 to 2.43) for CCS >2 for MACE (c-statistic = 0.77). Alternatively, setting the CCS < 2 as the referent group the adjusted HRs for MACE at 30 days for CCS = 2 was 2.83 (95%CI: 1.79 to 4.47) and for CCS > 2 was 4.91 (95%CI: 3.04 to 7.91) (Table S5). There was a statistically significant difference in the survival probabilities across the CCS groups within 365 days following discharge from ED (Figure 2, log-rank test *p*-value <0.0001 for primary outcome, see Supplement for Kaplan–Meier survival curves for all cause death (Figure S1), death/MI (Figure S2), and MACE (Figure S3) with number of patients at risk listed).

## 4. Discussion

Patients diagnosed with chest pain and discharged home from the ED in the province of Ontario, Canada have a 1% composite outcome (all-cause death, MI, UA hospital admission) at one month. This is in agreement with acceptable recommendations from emergency physician groups on miss rates in ED patients with possible ACS symptoms [23,24]. However, within this group of patients, the CCS can further aid in risk stratification. Specifically, a CCS < 2 represents a group that has nearly half the risk as compared to patients with a CCS = 2 in experiencing the composite outcome, whereas patients with a CCS > 2 represent a group that has nearly twice the risk as compared to the CCS = 2 group. This information and the calculation of the CCS can be readily and easily obtained from laboratory values at ED presentation which may further facilitate decision-making and patient discharge in the ED. Specifically, patients with a CCS > 2, may benefit from seeing a cardiologist following discharge from the ED to mitigate risk of future adverse events, and incorporation of such a score may affirm the need for appropriate care [27].

Equally important, a greater proportion of the population who are discharged home from the ED can be deemed to be at very low risk when using the CCS < 2 (>40%) as compared to applying a hs-cTn < 5 ng/L cutoff (<30%), thereby reassuring a larger population and possibly avoiding unnecessary medical intervention in these patients. The 5 ng/L cutoff has been reported as an appropriate cutoff to rule-out for both Roche and Abbott hs-cTn assays [7,8,25]. In this regard, a position paper of the Acute Cardiovascular Care Association which provided an update on the European Society of Cardiology task force report on the management of chest pain provided a management pathway where if the ECG is normal and the hs-cTn is below the limit of detection (e.g., hs-cTnT < 5 ng/L), discharge may be appropriate if the patient is pain free, differential diagnoses excluded and the HEART score is ≤ 3 [28]. Employing the CCS may further simplify this process and provide additional reassurance in this setting. The CCS may also be used in conjugation with serial measurements of hs-cTn, with the latter being important for assessing acute injury [3,17].

## 5. Limitations

This cohort study has some limitations that merit discussion. First, outcomes were assessed via administrative databases and thus there could be inaccuracies in diagnostic and physician billing codes. However, the approach undertaken here has been validated in other publications with others opining that outcomes via databases provide important real-world data [19,20,21,29]. Second, as the data are from multiple hospital sites throughout the province of Ontario, there are certainly pre-analytical, analytical and post-analytical variables that may affect high-sensitivity cardiac troponin result reporting and interpretation [2,9]. These variables can also affect creatinine and glucose, with a further complication being variation due to point of care testing and whole blood measurements [30]. Third, the CCS was retrospectively calculated for each patient and was not used in clinical decision-making. Fourth, we do not have complete information regarding the use of antiplatelet agents, anticoagulants, statins, and beta-blockers amongst the different subgroups of patients (i.e., CCS < 2, 2, >2). Fifth, our dataset does not contain information on ejection fraction and the type of stress testing in the study population. Accordingly, a prospective study assessing the CCS in this setting is required.

## 6. Conclusions

Application of the CCS for patients diagnosed with chest pain and discharged home from the ED may further identify low-risk (CCS < 2) and high-risk patients (CCS > 2), which may expedite decision-making in the ED and facilitate appropriate post-ED visit clinical care.

## Figures and Tables

**Table 1:** Comparison of baseline characteristics and crude outcomes by CCS (< 2, 2, > 2) for patients with a diagnosis of chest pain discharged home from the ED.

Variable	CCS < 2	CCS = 2	CCS > 2	Total	*p* Value
	*N* = 6404	*N* = 4336	*N* = 3936	*N* = 14,676	
**Demographics**					
Age in years, Median (IQR)	52 (46–60)	62 (52–71)	74 (64–82)	59 (50–71)	<0.001
Sex (F)	3805 (59.4%)	2396 (55.3%)	1890 (48.0%)	8091 (55.1%)	<0.001
**Past medical history, n (%)**					
Arrhythmia	100 (1.6%)	221 (5.1%)	578 (14.7%)	899 (6.1%)	<0.001
Congestive heart failure	129 (2.0%)	226 (5.2%)	841 (21.4%)	1196 (8.1%)	<0.001
Chronic obstructive pulmonary disorder	753 (11.8%)	772 (17.8%)	1042 (26.5%)	2567 (17.5%)	<0.001
Diabetes	819 (12.8%)	967 (22.3%)	1559 (39.6%)	3345 (22.8%)	<0.001
Hypertension	2308 (36.0%)	2472 (57.0%)	3138 (79.7%)	7918 (54.0%)	<0.001
Myocardial infarction	171 (2.7%)	209 (4.8%)	491 (12.5%)	871 (5.9%)	<0.001
Peripheral vascular disease	70 (1.1%)	129 (3.0%)	515 (13.1%)	714 (4.9%)	<0.001
Renal disease	6 (0.1%)	15 (0.3%)	171 (4.3%)	192 (1.3%)	<0.001
Stroke	35 (0.5%)	31 (0.7%)	88 (2.2%)	154 (1.0%)	<0.001
Unstable angina	79 (1.2%)	154 (3.6%)	286 (7.3%)	519 (3.5%)	<0.001
Percutaneous coronary intervention	190 (3.0%)	243 (5.6%)	375 (9.5%)	808 (5.5%)	<0.001
Coronary artery bypass grafting	28 (0.4%)	61 (1.4%)	115 (2.9%)	204 (1.4%)	<0.001
Echocardiography	2251 (35.1%)	2043 (47.1%)	2577 (65.5%)	6871 (46.8%)	<0.001
Cardiac catheterization	395 (6.2%)	504 (11.6%)	819 (20.8%)	1718 (11.7%)	<0.001
Stress testing	2008 (31.4%)	1788 (41.2%)	1822 (46.3%)	5618 (38.3%)	<0.001
**Physician follow-up 30 days following ED discharge, n (%)**					
Cardiology	1767 (27.6%)	1525 (35.2%)	1598 (40.6%)	4890 (33.3%)	<0.001
General/Family physician	2358 (36.8%)	1552 (35.8%)	1474 (37.4%)	5384 (36.7%)	
None	2279 (35.6%)	1259 (29.0%)	864 (22.0%)	4402 (30.0%)	
**All-cause mortality or hospitalization for MI/UA, n (%)**					
30 days	18 (0.3%)	37 (0.9%)	92 (2.3%)	147 (1.0%)	<0.001
90 days	28 (0.4%)	48 (1.1%)	182 (4.6%)	258 (1.8%)	<0.001
365 days	82 (1.3%)	146 (3.4%)	437 (11.1%)	665 (4.5%)	<0.001

**Table 2:** Cox proportional hazard model estimates for the primary outcome of all-cause mortality, MI, and UA at 30 days, 90 days, and 365 days.

Time of Outcome Assessment	Model *	CCS Category	Hazard Ratio (95% CI)
30 days	1	CCS < 2	0.33 (0.19–0.58)
		CCS > 2	2.76 (1.89–4.05)
	2	CCS < 2	0.38 (0.21–0.67)
		CCS > 2	2.31 (1.53–3.47)
	3	CCS < 2	0.40 (0.23–0.72)
		CCS > 2	2.05 (1.35–3.13)
90 days	1	CCS < 2	0.39 (0.25–0.62)
		CCS > 2	4.24 (3.09–5.83)
	2	CCS < 2	0.49 (0.30–0.79)
		CCS > 2	3.26 (2.35–4.56)
	3	CCS < 2	0.51 (0.32–0.82)
		CCS > 2	2.80 (1.98–3.97)
365 days	1	CCS < 2	0.38 (0.29–0.49)
		CCS > 2	3.44 (2.85–4.15)
	2	CCS < 2	0.56 (0.43–0.75)
		CCS > 2	2.20 (1.80–2.67)
	3	CCS < 2	0.59 (0.45–0.78)
		CCS > 2	1.76 (1.43–2.17)

* Model 1 unadjusted; Model 2 adjusted for age and sex; Model 3 adjusted for age, sex, prior history of arrhythmia, heart failure, diabetes, hypertension, MI, peripheral vascular disease, renal disease, stroke and UA. Reference group is CCS = 2 and all *p*-values are <0.01.

## Supplementary Materials

The following are available online at https://www.mdpi.com/2077-0383/9/9/2948/s1, Figure S1: Kaplan Meier survival curves for all-cause death over 365 days stratified by CCS < 2, CCS = 2, and CCS > 2, Figure S2: Kaplan Meier survival curves for death/MI over 365 days stratified by CCS < 2, CCS = 2, and CCS > 2, Figure S3: Kaplan Meier survival curves for MACE over 365 days stratified by CCS < 2, CCS = 2, and CCS > 2, Table S1: Comparison of baseline characteristics and crude outcomes by CCS (< 2, 2, > 2) for patients with a diagnosis of chest pain discharged home from the ED from Cohort 1, Table S2: Comparison of baseline characteristics and crude outcomes by CCS (< 2, 2, > 2) for patients with a diagnosis of chest pain discharged home from the ED from Cohort 2, Table S3: Cox proportional hazard model estimates for CCS, all-cause mortality plus hospitalization for MI/UA for Cohort 1 with hs-cTnI, Table S4: Cox proportional hazard model estimates for CCS, all-cause mortality plus hospitalization for MI/UA for Cohort 2 with hs-cTnT, Table S5: Cox proportional hazard model estimates (model 3) for the secondary outcome of MACE (defined as the composite of death/MI/UA/PCI or CABG) at 30 days, 90 days, and 365 days with CCS < 2 as the reference group for the study population (*n* = 14,676).
