# Supplementary material for: Risk Stratification for Patients with Chest Pain Discharged Home from the Emergency Department
Source: J Clin Med. 2020 Sep 12;9(9):2948. doi: 10.3390/jcm9092948 (PMC7565964; doi:10.3390/jcm9092948)
Supplement: Supplementary file 1 [file jcm-09-02948-s001.pdf]

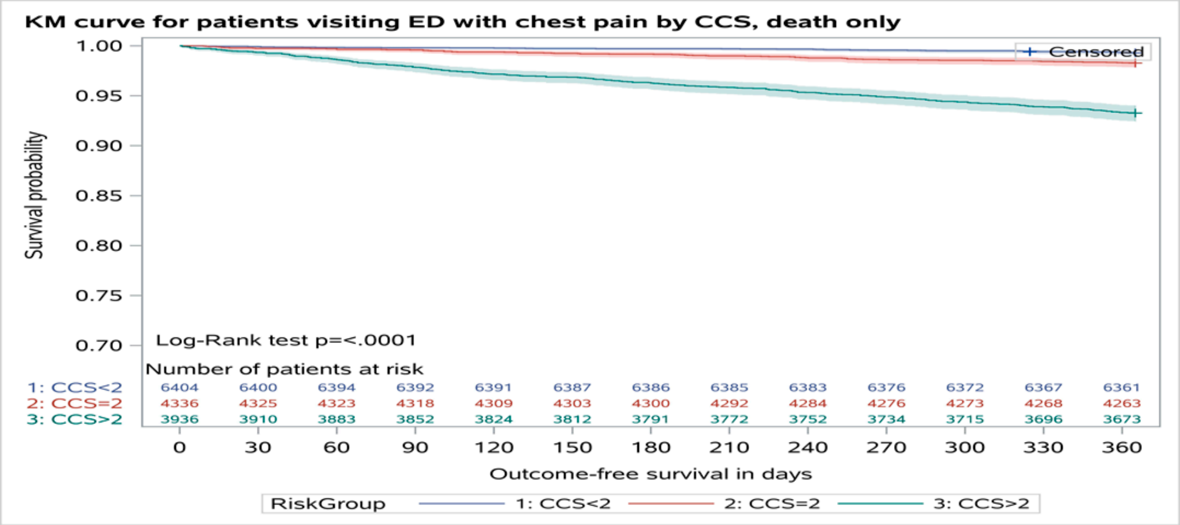

Figure S1. Kaplan Meier survival curves for all-cause death over 365 days stratified by CCS < 2, CCS = 2, and CCS >2.

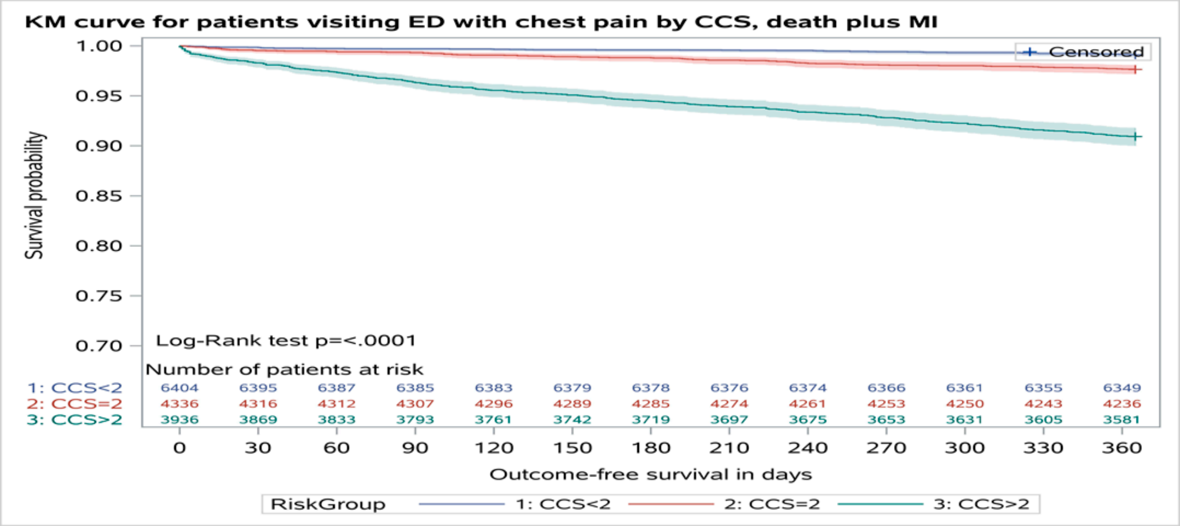

Figure S2. Kaplan Meier survival curves for death/MI over 365 days stratified by CCS < 2, CCS = 2, and CCS >2.

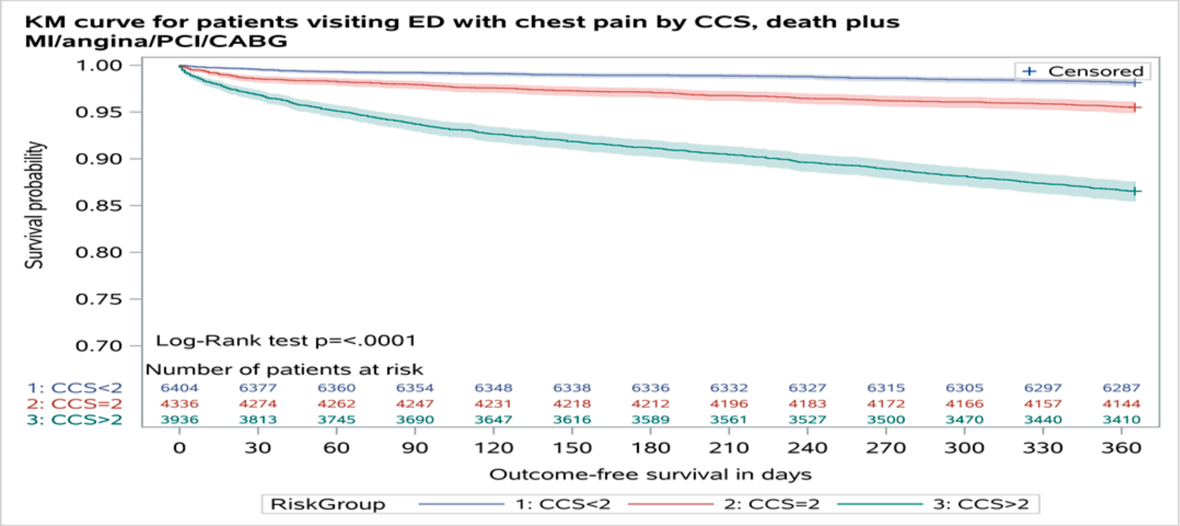

Figure S3. Kaplan Meier survival curves for MACE over 365 days stratified by CCS < 2, CCS = 2, and CCS >2.

Table S1. Comparison of baseline characteristics and crude outcomes by CCS (< 2, 2, > 2) for patients with a diagnosis of chest pain discharged home from the ED from Cohort1.

Variable	CCS<2	CCS=2	CCS>2	P-Value
	N=456	N=457	N=454	
Age, Median (IQR)	53 (48-62)	65 (54-76)	76 (66-82)	<.001
Sex (F)	260 (57.0%)	285 (62.4%)	232 (51.1%)	0.003
**Past medical history, n (%)**				
Arrhythmia	16 (3.5%)	43 (9.4%)	101 (22.2%)	<.001
Congestive heart failure	24 (5.3%)	41 (9.0%)	123 (27.1%)	<.001
Chronic obstructive pulmonary disorder	89 (19.5%)	104 (22.8%)	126 (27.8%)	0.013
Diabetes	64 (14.0%)	129 (28.2%)	190 (41.9%)	<.001
Hypertension	192 (42.1%)	309 (67.6%)	365 (80.4%)	<.001
Myocardial infarction	20 (4.4%)	28 (6.1%)	66 (14.5%)	<.001
Peripheral vascular disease	8 (1.8%)	25 (5.5%)	69 (15.2%)	<.001
Renal disease	<=5	7 (1.5%)	25 (5.5%)	<.001
Stroke	9 (2.0%)	6 (1.3%)	17 (3.7%)	0.043
Unstable angina	11 (2.4%)	26 (5.7%)	24 (5.3%)	0.033
Percutaneous coronary intervention	10 (2.2%)	20 (4.4%)	23 (5.1%)	0.064
Coronary artery bypass grafting	<=5	11 (2.4%)	15 (3.3%)	0.008
Echocardiography	168 (36.8%)	233 (51.0%)	311 (68.5%)	<.001
Cardiac catheterization	32 (7.0%)	62 (13.6%)	89 (19.6%)	<.001
Permanent pacemaker	<=5	<=5	16 (3.5%)	<.001
Implantable cardioverter-defibrillator	<=5	<=5	8 (1.8%)	0.005
Stress testing	137 (30.0%)	165 (36.1%)	159 (35.0%)	0.119
**Physician follow-up 30 days following ED discharge, n (%)**				
Cardiology	29 (6.4%)	45 (9.8%)	63 (13.9%)	0.001
General/Family physician	224 (49.1%)	234 (51.2%)	230 (50.7%)	
None	203 (44.5%)	178 (38.9%)	161 (35.5%)	
**All- cause mortality or hospitalization for MI/angina, n (%)**				
30 days	<=5	<=5	8 (1.8%)	0.055
90 days	<=5	<=5	21 (4.6%)	<.001
1 year	11 (2.4%)	26 (5.7%)	60 (13.2%)	<.001
**Laboratory Values**				
Serum creatinine, umol/L, Median (IQR)	70 (64-77)	76 (69-89)	90 (75-114)	<.001
Glucose, mmol/L, Mean ± SD	5.74 ± 1.80	6.92 ± 2.84	7.56 ± 2.74	<.001
hs-cTnI, ng/L, Median (IQR)	2 (1-3)	3 (2-5)	9 (6-16)	<.001
Estimated glomerular filtration rate, mL/min/1.73 m^2, Median (IQR)	94 (86-101)	78 (66-86)	64 (47-78)	<.001

Table S2. Comparison of baseline characteristics and crude outcomes by CCS (< 2, 2, > 2) for patients with a diagnosis of chest pain discharged home from the ED from Cohort2.

Variable	CCS<2	CCS=2	CCS>2	P-Value
	N=5,948	N=3,879	N=3,482	
Age, Median (IQR)	52 (46-59)	61 (52-71)	74 (63-82)	<.001
Sex (F)	3,545 (59.6%)	2,111 (54.4%)	1,658 (47.6%)	<.001
**Past medical history, n (%)**				
Arrhythmia	84 (1.4%)	178 (4.6%)	477 (13.7%)	<.001
Congestive heart failure	105 (1.8%)	185 (4.8%)	718 (20.6%)	<.001
Chronic obstructive pulmonary disorder	664 (11.2%)	668 (17.2%)	916 (26.3%)	<.001
Diabetes	755 (12.7%)	838 (21.6%)	1,369 (39.3%)	<.001
Hypertension	2,116 (35.6%)	2,163 (55.8%)	2,773 (79.6%)	<.001
Myocardial infarction	151 (2.5%)	181 (4.7%)	425 (12.2%)	<.001
Peripheral vascular disease	62 (1.0%)	104 (2.7%)	446 (12.8%)	<.001
Renal disease	<=5	8 (0.2%)	146 (4.2%)	<.001
Stroke	26 (0.4%)	25 (0.6%)	71 (2.0%)	<.001
Unstable angina	68 (1.1%)	128 (3.3%)	262 (7.5%)	<.001
Percutaneous coronary intervention	180 (3.0%)	223 (5.7%)	352 (10.1%)	<.001
Coronary artery bypass grafting	26 (0.4%)	50 (1.3%)	100 (2.9%)	<.001
Echocardiography	2,083 (35.0%)	1,810 (46.7%)	2,266 (65.1%)	<.001
Cardiac catheterization	363 (6.1%)	442 (11.4%)	730 (21.0%)	<.001
Permanent pacemaker	<=5	24 (0.6%)	54 (1.6%)	<.001
Implantable cardioverter-defibrillator	<=5	6 (0.2%)	12 (0.3%)	<.001
Stress testing	1,871 (31.5%)	1,623 (41.8%)	1,663 (47.8%)	<.001
**Physician follow-up 30 days following ED discharge, n (%)**				
Cardiology	1,738 (29.2%)	1,480 (38.2%)	1,535 (44.1%)	<.001
General/Family physician	2,134 (35.9%)	1,318 (34.0%)	1,244 (35.7%)	
None	2,076 (34.9%)	1,081 (27.9%)	703 (20.2%)	
**All- cause mortality or hospitalization for MI/angina, n (%)**				
30 days	17 (0.3%)	33 (0.9%)	84 (2.4%)	<.001
90 days	23 (0.4%)	43 (1.1%)	161 (4.6%)	<.001
365 days	71 (1.2%)	120 (3.1%)	377 (10.8%)	<.001
**Laboratory Values**				
Creatinine, umol/L, Median (IQR)	66 (58-77)	77 (68-89)	89 (75-107)	<.001
Glucose, mmol/L, Mean ± SD	5.88 ± 1.84	6.68 ± 2.35	7.49 ± 2.94	<.001
hs-cTnT, ng/L, Median (IQR)	5 (3-6)	7 (5-13)	14 (12-24)	<.001
Estimated glomerular filtration rate, mL/min/1.73 m^2, Median (IQR)	97 (91-104)	81 (71-89)	67 (51-80)	<.001

Table S3. Cox proportional hazard model estimates for CCS, all-cause mortality plus hospitalization for MI/UA for Cohort 1 with hs-cTnI.

Time of outcome assessment	Model*	CCS category	Hazard ratio (95% CI)
30 days	1	CCS < 2	0.25 (0.03-2.24)
		CCS > 2	2.02 (0.61-6.72)
	2	CCS < 2	0.51 (0.05-4.91)
		CCS > 2	1.24 (0.35-4.33)
	3	CCS < 2	0.57 (0.06-5.49)
		CCS > 2	1.34 (0.38-4.76)
90 days	1	CCS < 2	1.00 (0.29-3.45)
		CCS > 2	4.29 (1.62-11.38)
	2	CCS < 2	1.85 (0.51-6.73)
		CCS > 2	2.82 (1.03-7.71)
	3	CCS < 2	1.96 (0.53-7.21)
		CCS > 2	2.48 (0.89-6.86)
365 days	1	CCS < 2	0.42 (0.21-0.85)
		CCS > 2	2.42 (1.53-3.84)
	2	CCS < 2	0.77 (0.37-1.61)
		CCS > 2	1.55 (0.96-2.51)
	3	CCS < 2	0.83 (0.39-1.74)
		CCS > 2	1.32 (0.81-2.16)

* Model 1 unadjusted; Model 2 adjusted for age and sex; Model 3 adjusted for age, sex, prior history of arrhythmia, heart failure, diabetes, hypertension, MI, peripheral vascular disease, renal disease, stroke and UA. Reference group is CCS = 2.

Table S4. Cox proportional hazard model estimates for CCS, all-cause mortality plus hospitalization for MI/UA for Cohort 2 with hs-cTnT.

Time of outcome assessment	Model*	CCS category	Hazard ratio (95% CI)
30 days	1	CCS < 2	0.34 (0.19-0.60)
		CCS > 2	2.86 (1.91-4.28)
	2	CCS < 2	0.37 (0.21-0.68)
		CCS > 2	2.49 (1.62-3.84)
	3	CCS < 2	0.40 (0.22-0.72)
		CCS > 2	2.14 (1.37-3.34)
90 days	1	CCS < 2	0.35 (0.21-0.58)
		CCS > 2	4.24 (3.03-5.93)
	2	CCS < 2	0.42 (0.25-0.70)
		CCS > 2	3.37 (2.36-4.83)
	3	CCS < 2	0.43 (0.26-0.73)
		CCS > 2	2.87 (1.99-4.16)
1 year	1	CCS < 2	0.38 (0.28-0.51)
		CCS > 2	3.65 (2.97-4.48)
	2	CCS < 2	0.55 (0.41-0.75)
		CCS > 2	2.38 (1.91-2.96)
	3	CCS < 2	0.58 (0.43-0.78)
		CCS > 2	1.89 (1.50-2.37)

* Model 1 unadjusted; Model 2 adjusted for age and sex; Model 3 adjusted for age, sex, prior history of arrhythmia, heart failure, diabetes, hypertension, MI, peripheral vascular disease, renal disease, stroke and UA. Reference group is CCS =2.

Table S5. Cox proportional hazard model estimates (model 3) for the secondary outcome of MACE (defined as the composite of death/MI/UA/PCI or CABG) at 30 days, 90 days, and 365 days with CCS < 2 as the reference group for the study population (n=14,676).

Time of outcome assessment	CCS category	Hazard ratio (95% CI)*
30 days	CCS = 2	2.83 (1.79-4.47)
	CCS > 2	4.91 (3.04-7.91)
90 days	CCS = 2	2.26 (1.58-3.22)
	CCS > 2	5.27 (3.69-7.54)
365 days	CCS = 2	1.74 (1.38-2.21)
	CCS > 2	3.19 (2.51-4.07)

* all p-values are <0.01.
